# Biostimulant Action of Protein Hydrolysates: Unraveling Their Effects on Plant Physiology and Microbiome

**DOI:** 10.3389/fpls.2017.02202

**Published:** 2017-12-22

**Authors:** Giuseppe Colla, Lori Hoagland, Maurizio Ruzzi, Mariateresa Cardarelli, Paolo Bonini, Renaud Canaguier, Youssef Rouphael

**Affiliations:** ^1^Department of Agricultural and Forestry Sciences, University of Tuscia, Viterbo, Italy; ^2^Department of Horticulture and Landscape Architecture, Purdue University, West Lafayette, IN, United States; ^3^Department for Innovation in Biological, Agrofood and Forest Systems, University of Tuscia, Viterbo, Italy; ^4^Consiglio per la Ricerca in Agricoltura e l’Analisi dell’Economia Agraria, Centro di Ricerca Orticoltura e Florovivaismo, Pontecagnano, Italy; ^5^NGA Laboratory, Tarragona, Spain; ^6^Nixe, Valbonne, France; ^7^Department of Agricultural Sciences, University of Naples Federico II, Portici, Italy

**Keywords:** abiotic stress, amino acids, enzymatic hydrolysis, microbial inoculants, peptides, product quality, physiological mechanisms, sustainable agriculture

## Abstract

Plant-derived protein hydrolysates (PHs) have gained prominence as plant biostimulants because of their potential to increase the germination, productivity and quality of a wide range of horticultural and agronomic crops. Application of PHs can also alleviate the negative effects of abiotic plant stress due to salinity, drought and heavy metals. Recent studies aimed at uncovering the mechanisms regulating these beneficial effects indicate that PHs could be directly affecting plants by stimulating carbon and nitrogen metabolism, and interfering with hormonal activity. Indirect effects could also play a role as PHs could enhance nutrient availability in plant growth substrates, and increase nutrient uptake and nutrient-use efficiency in plants. Moreover, the beneficial effects of PHs also could be due to the stimulation of plant microbiomes. Plants are colonized by an abundant and diverse assortment of microbial taxa that can help plants acquire nutrients and water and withstand biotic and abiotic stress. The substrates provided by PHs, such as amino acids, could provide an ideal food source for these plant-associated microbes. Indeed, recent studies have provided evidence that plant microbiomes are modified by the application of PHs, supporting the hypothesis that PHs might be acting, at least in part, via changes in the composition and activity of these microbial communities. Application of PHs has great potential to meet the twin challenges of a feeding a growing population while minimizing agriculture’s impact on human health and the environment. However, to fully realize the potential of PHs, further studies are required to shed light on the mechanisms conferring the beneficial effects of these products, as well as identify product formulations and application methods that optimize benefits under a range of agro-ecological conditions.

## Introduction

In the coming years, agriculture must meet the twin challenge of feeding a growing global population, while simultaneously minimizing agriculture’s impact on human health and the environment (Searchinger, 2013). To meet global demand several solutions have been proposed, that focus on breeding varieties with greater yield potential, however, this one-size-fits-all solution leads to limited benefits, especially given that limits of the genetic potential of staple crops have almost been reached. Alternatively, it has been hypothesized that to increase the reliability and stability of agricultural crop yield, optimizing crop management and improving resource use efficiency (i.e., fertilizers and water) under different agro-ecological conditions, holds the key to sustainably increase yield across different environments and years. In other words produce *‘more with less.’*

An innovative technology with promising application potential in confronting these critical challenges entails the use of protein hydrolysates (PHs). Application of PHs as biostimulants on a wide range of horticultural and agronomic crops has been acclaimed. PHs are *‘mixtures of polypeptides, oligopeptides and amino acids that are manufactured from protein sources using partial hydrolysis’* (Schaafsma, 2009). They are available as liquid extracts or in soluble powder and granular form, and may be side-dressed near the root or applied as foliar sprays (Colla et al., 2015a). PHs are mainly produced by chemical (acid and alkaline hydrolysis), thermal and enzymatic hydrolysis of a wide range of both animal wastes and plant biomass (Colla et al., 2015a; du Jardin, 2015; Halpern et al., 2015). Animal residues include animal epithelial or connective tissues such as leather by-products, blood meal, fish by-products, chicken feathers and casein, whereas biomass of plant origin includes legume seeds, alfalfa hay, corn wet-milling and vegetable by-products (Colla et al., 2015a). In particular, PHs coming from by-products of vegetables and the corn wet-milling industry are gaining popularity among the scientific community and commercial enterprises, since they could represent a sustainable, economical and eco-friendly solution to the problem of waste disposal (Pecha et al., 2012; Baglieri et al., 2014). Currently, most of the market for PHs biostimulants accounts for animal-derived proteins procured through acid hydrolysis, with the remainder coming from enzymatic hydrolysis of plant-derived proteins (Colla et al., 2015a). On a global scale, most of the PHs for agricultural use are produced from companies located in Italy, Spain, United States, China and India. Some of these companies in Europe and East Asia were developed by leather/meat industries as a way to valorize their by-products through the production of biostimulants and fertilizers. Moreover, in the last years some companies introduced plant-derived PHs in the United States, European and Asian market; these plant-derived PHs are gaining greater acceptance by farmers due to their richness in bioactive compounds and their great efficacy in enhancing crop performances.

In many cases, PHs have been demonstrated to play key roles as biostimulants through the modulation of plant molecular and physiological processes that trigger growth, increase yield and alleviate the impact of abiotic stress on crops (Calvo et al., 2014; Yakhin et al., 2017). These include salinity, heavy metal, thermal, nutrient stress, and water stress (Botta, 2013; Cerdán et al., 2013; Colla et al., 2013, 2014; Ertani et al., 2013; Lucini et al., 2015; Rouphael et al., 2017a). Direct effects of PHs on plants include stimulation of carbon and nitrogen metabolism, as well as regulation of N uptake mediated by key enzymes involved in the N assimilation process and regulation of the activity of three enzymes involved in the tricarboxylic acid cycle (citrate synthase, isocitrate dehydrogenase and malate dehydrogenase) (Colla et al., 2015a; du Jardin, 2015; Nardi et al., 2016). PHs could also interfere with hormonal activities, due to the presence of bioactive peptides (Colla et al., 2014, 2015a). Several studies have demonstrated that many commercial products obtained from PHs elicited hormone-like activities (auxin and gibberellins), promoting root and shoot growth, and thus crop productivity (Ertani et al., 2009; Matsumiya and Kubo, 2011; Colla et al., 2014; Lucini et al., 2015).

In addition, to the direct effect of PHs, indirect effects on growth and plant nutrition have been also demonstrated when PHs were applied to soils and plants (du Jardin, 2015). Foliar and root applications have been shown to enhance the uptake and use efficiency of both macro and micronutrients (Ertani et al., 2009; Colla et al., 2015a; Halpern et al., 2015). Improved nutrient uptake performance of PH-treated plants has been mostly associated with modifications of root architecture (density, length and number of lateral roots), as well as to an increase of nutrient availability in the soil solution resulting from complexation of nutrients by peptides and amino acids, and enhanced microbial activity (Colla et al., 2015a; du Jardin, 2015). In addition to the positive effects of PH-treated plants, there are several authors (Ruiz et al., 2000; Cerdán et al., 2009; Lisiecka et al., 2011) reporting phytotoxicity effects as well as suppression of growth related to the use of animal-derived PHs of fruiting crops. This phenomenon is known as ‘general amino acid inhibition’ (Bonner and Jensen, 1997) and it is due to excessive leaf uptake of free-amino acids, which causes intracellular amino acid imbalance, energy drain due to active transport of amino acids, inhibition of nitrate uptake, and increase of cell susceptibility to apoptosis.

Despite the efforts of scientists to understand the biostimulant properties of PHs, knowledge about target metabolic pathways, mechanisms of action elicited by the application of PHs is far from unraveled. Moreover, the biostimulant action of PHs can vary depending on their origin and characteristics, species, cultivars, phenological stages, growing conditions, concentration, time and mode (leaf versus root) of application, solubility, and leaf permeability (Colla et al., 2015a). The penetration of active ingredients (amino acids and peptides) into internal structures of PH-treated plants is crucial since PHs based biostimulant are usually foliarly applied (Colla et al., 2015a; Yakhin et al., 2017).

The biostimulant activity observed in response to the application of PHs could be acting, at least in part, indirectly through a microbially mediated enhancement of plant health (Colla et al., 2014). It is now commonly accepted that microbes can improve plant fitness by altering physiological and development processes, resulting in greater nutrient and water uptake as well as enhanced resilience against environmental stressors (Philippot et al., 2013). Many of these interactions have been found to occur in the rhizosphere, which encompasses a limited soil volume confined to and affected by the root system. More recently, microbes that promote growth and help plants to withstand biotic and abiotic stress have also been found in the phyllosphere, which covers plant leaf surfaces. It has been estimated that the number of microbial cells living on and within plant tissues outnumbers plant cells, and this community of microorganisms is now commonly referred to as part of the 2nd genome of the plant, or its microbiome (Berendsen et al., 2012). The organic molecules in PHs could be adopted as a source of carbon and nitrogen for the microbes residing in the rhizosphere and phyllosphere of plants. Moreover, microbes are generally more competitive for amino acids than plants (Moe, 2013), indicating that much of the organic materials provided by PHs could be utilized or altered by microbes before they can directly influence or be taken up by plants. If this proves to be correct, understanding how to modify the plant microbiome with PHs has potential to enhance their benefits and further improve plant productivity.

The aim of the current review is to provide an updated scientific overview of the effects of PHs on growth, productivity, and quality of agricultural commodities; moreover, it sheds light on the possible modes of action and mechanisms mediating these effects. The impact of PHs application on the primary and secondary metabolism, and physiology, the resilience to adverse chemical soil conditions and environmental stresses, as well as the effects of PHs on the plant microbiome are also covered.

## Chemical Characteristics

Protein hydrolysates contain mainly peptides and free amino acids (Calvo et al., 2014). PHs can also contain carbohydrates and negligible quantities of mineral elements, phenols, phytohormones and other organic compounds (Ertani et al., 2014; Colla et al., 2015a). Chemical characteristics of PHs vary depending on source of proteins (e.g., collagen from leather by-products, fish by-products, legume seeds, alfa-alfa biomass), and production process (chemical and/or enzymatic hydrolysis). Collagen-derived PHs composition is dominated by amino acids like glycine and proline as well as aspartic and glutamic acids in legume-derived and fish-derived PHs (Ertani et al., 2009, 2013; Chalamaiah et al., 2012; Colla et al., 2015a). Moreover, collagen derived-PHs typically contain significant amounts of hydroxyproline and hydroxylysine, which can be used as markers for this type of PHs (Colla et al., 2015a).

Animal derived-PHs are usually produced through chemical hydrolysis with the use of acids (hydrochloric and sulphuric acid) at high temperature (>121°C) and pressure (>220.6 kPa). Because acid hydrolysis is very aggressive, the resulting product is composed of a large amount of free amino acids and to a lesser extent by soluble peptides. During the acid hydrolysis some amino acids like tryptophan, cysteine, serine and threonine are partially or totally destroyed and many other amino acids are converted from the L-form to D-form (racemisation) thus losing their biological activity (Colla et al., 2015a). Since plant derived-PHs are produced through a more gentle method (enzymatic hydrolysis using proteolytic enzymes and temperature below 60°C), the resulting PHs contain higher peptides:free amino acids ratio, and proportion of L-amino acids in comparison with those obtained by chemical hydrolysis. The peptides molecular weight varies from several hundred to thousands of Daltons, with low molecular weight peptides being more biologically active (Quartieri et al., 2002). Biologically active peptides have been isolated and chemically characterized from PHs, especially those derived from plant materials. For instance, a short peptide (12 amino acids) called ‘root hair promoting peptide’ has been identify in a soybean-derived PHs (Matsumiya and Kubo, 2011) and in the commercial legume derived-PHs ‘Trainer^®^’. Many other bioactive peptides acting as signaling molecules in plant defense, growth, and development have been discovered in plant tissues (e.g., systemins, phytosulfokines, clavata3) (Ryan et al., 2002), and may also been present in PHs.

## Effects on Physiological and Agronomic Traits of Crops

### Germination and Seedling Growth

Several technologies have been proposed to enhance sowing and seedling establishment under a wide range of environmental conditions. These technologies include seed conditioning and priming as well as seed coating. Seed coated with hydrophilic materials and hydro-absorbers can protect young seedlings from pests, diseases, fungi and low temperature (Gorim and Asch, 2012). Seed coatings may also contain macro and micronutrients (Farooq et al., 2012), herbicides (Rushing et al., 2013), growth regulators (Halmer, 2004) and beneficial microorganisms (Colla et al., 2015b).

In recent years, several commercial enterprises were interested in whether the biostimulant material could be applied as a component of a seed coating blend. In a recent study, Amirkhani et al. (2016) and co-workers showed that broccoli (*Brassica oleracea* L.) seed coating formulations (soy flour/cellulose fiber/diatomaceous earth, termed as SCD) using soy flour at a concentration of 10% had greater seedling shoot and root growth compared to uncoated seeds. In contrast, germination was negatively affected by seed coating with SCD likely due to the fact that the treatment binder may have acted as a barrier for water uptake and gas exchange (Mucke, 1988; Hill, 1999). However, after 1 month in the greenhouse, the fresh and dry biomass, plant height, leaf area, Soil Plant Analyses Development (SPAD) index, as well as total nitrogen of broccoli plantlets were always higher in plants with seed coatings of 30, 40, and 50% soy flour in comparison to the uncoated control. The authors concluded that using soy flour as seed coating materials improved several growth characteristics by triggering nitrogen uptake, assimilation and translocation, by enhancing some key enzymes involved in nitrogen metabolism. Colla et al. (2014) conducted laboratory bioassays using the PH ‘Trainer^®^’ containing 31% of soluble peptides and free amino acids. In their study, treatment of detached corn (*Zea mays* L.) coleoptiles with the plant-derived PH having elicited an accelerated coleoptile elongation in comparison to the non-treated control (i.e., deionized water). Moreover, no significant effects were recorded on coleoptile elongation rate among the four PH concentrations studied (0.375, 0.75, 1.5, and 3 ml/L) and IAA treatment. The authors concluded that a significant auxin-like activity occurred using the plant-derived PH ‘Trainer^®^,’ likely due to the presence of tryptophan, a major precursor for IAA biosynthesis and bioactive peptides. Like auxins, gibberellins are known to improve cell elongation and function as chemical signals promoting the biosynthesis of α-amylase, which is important during germination (Parrado et al., 2008). The application of PH ‘Trainer^®^’ at four doses (0.375, 0.75, 1.5, and 3 ml/L) enhanced the shoot length of gibberellin-dwarf pea (*Pisum sativum* L.) plants by 33% compared with the control treatment, with no significant differences between the four dose rates, providing clear evidence of gibberellin-like activity (Colla et al., 2014). The results of Colla et al. (2014) confirm a previous report by Ghosh et al. (2010), who provided evidence that wheat peptides mimic hormonal activity like that of gibberellins.

In addition to the beneficial role of plant-derived PHs on plant growth, the positive effects of animal-derived protein application also have been demonstrated. Gelatin, an animal-derived protein, applied as capsules placed near the seeds, has been shown to act as a biostimulant on greenhouse-grown cucumber (*Cucumis sativus* L.). Application of these gelatin capsules increased fresh and dry weight biomass, leaf area and nitrogen content of 2-week old plants compared with seeds sown without gelatin capsules (Wilson et al., 2015). Changes in plant biomass and nitrogen in response to the gelatin capsules were correlated with an up-regulation of both amino acids and N transporter genes and the xenobiotic detoxification system. The authors concluded that these genes, and their possible transcriptional regulation through the two transcription factors, could be an important mechanism regulating improved plant growth following gelatin seed treatment. The use of collagen hydrolysate in wheat seed treatment showed a stimulation of seed metabolism by increasing endogenous gibberellic acid, and an enhancement of emergence and seedling biomass, and a reduction of abnormal seedlings (Gaidau et al., 2015). Furthermore, Gaidau et al. (2013) showed that cereal seed treatments with collagen-based hydrolysate mixes with fungicides and insecticides reduced pesticide needs, with diminished environmental impact and reduced cost of seed treatment.

Similarly, a vegetal-PH based product (BioST VPH, Albaugh, LLC, Valdosta, GA, United States) containing a root hair promoting peptide has been successfully used as a seed treatment to stimulate early root growth, crop stress tolerance and promote adhesion of fungicides/micronutrients on seed surface in corn (Zea mays L.) and soybean [*Glycine max* (L.) Merr.] (Bonini et al., 2017).

Moreover, the use of these compounds as seed treatments can provide additional benefits, such as the reduction of dust formation and prevention of microbial inoculant detachment from the seed surface during handling. The adhesive properties of PHs are primarily related to the ‘sticky’ small cationic peptides. A recent patent (n. 201531523/3 presented on October 22, 2015) proposed by Agrotecnologías Naturales SL (Tarragona, Spain) showed that a soybean-derived PH was able to more than double the number of polyethylene microspheres (having 75 – 90 μm of diameter, and used as substitute for arbuscular mycorrhizal fungi spores) that stuck to the seed surface of wheat (*Triticum aestivum* L.), corn (Zea mays L.) and soybean [*Glycine max* (L.) Merr.] in comparison with water. Moreover, mechanical vibration of coated seeds, showed that adding the soybean-derived PH to the microsphere/water suspension increased the adhesion strength of the microspheres by about 96, 36, and 21% in wheat, corn and soybean seeds, respectively.

### Plant Growth and Productivity

Several experimental studies testing the action of PHs under both open-field and controlled conditions, have demonstrated that they stimulate shoot and root biomass, resulting in increased productivity of several crops such as corn, kiwifruit, lettuce, lily, papaya, passiofruit, pepper and tomato (Schiavon et al., 2008; Ertani et al., 2009; Colla et al., 2014, 2015a, 2017; Halpern et al., 2015; Nardi et al., 2016). Foliar application of animal and plant-derived PHs has also been shown to promote the vegetative growth and yield of several fruit trees (Colla et al., 2015a). For instance, papaya (*Carica papaya* L.) plants sprayed at a 30-day interval with animal-derived PH ‘Siapton’ (i.e., increased crop productivity by 22% in comparison to the untreated control treatment (Morales-Pajan and Stall, 2004). Similarly, in banana (*Musa* spp.), foliar spray of banana plants with hydrolyzed poultry feather processing waste, condensed the time to harvest by 4 weeks and enhanced several yield components such as the number of hands per bunch and the mean bunch weight in comparison to untreated plants. Stimulation of banana crop performance and growth in these experiments was correlated with greater reducing sugars and chlorophyll concentrations in the PH-treated plants.

Greenhouse applications of an animal-derived PH ‘Siapton,’ and carob germ hydrolysate enhanced both plant height as well as number of flowers per plant in tomato (*Solanum lycopersicum* L.) compared with untreated plants, though only those sprayed with carob germ hydrolysate improved the number of fruit per plant after 18 weeks (Parrado et al., 2008). In greenhouse tomatoes, Koukounararas et al. (2013) showed that root or foliar spray of a PH commercial product, Amino 16^®^, containing 11.3% L-amino acids, enhanced yield by increasing both fruit number and mean weight, irrespective of fertilization rate. Similarly, foliar applications of the legume-derived PH ‘Trainer^®^’ at 5.0 ml L^-1^ increased marketable yield of two fresh-market tomato cultivars by modulating yield components differently depending on the cultivar, with higher number of fruits in Akyra and greater fruit mean weight in Sir Elyan (Rouphael et al., 2017b). Increasing the dose of plant-derived PH ‘Trainer^®^’ from 0 to 10 ml/L caused significant increase in shoots, root and total dry biomass, greenness readings as well as leaf N content (Colla et al., 2014) by 19.5, 27.5, 20.5, 15.1, and 21.5%, respectively, but there were no differences observed between the biostimulant at concentrations (5 and 10 ml/L). In the same study, enhancement in growth and nitrogen metabolism in PH-treated tomato plants was attributed to stimulation of nitrogen uptake and assimilation, which may improve net CO_2_ assimilation and enhance the translocation of photosynthates (i.e., soluble sugars) via the phloem to potential sinks (Ertani et al., 2009). A presumed mechanism involved in the stimulation of nitrogen assimilation in response to PHs, is the increase in the activity of two key enzymes, nitrate reductase and glutamine synthetase (Ertani et al., 2009). Another possible mechanism involved in the biostimulant effect of PH-treated plants could be related to stimulation of a more vigorous root system, which may enhance the efficiency of water and nutrient uptake, thus boosting crop yield. In a recent rooting experiment of tomato cuttings, Colla et al. (2014) observed that root dry weight, root length and surface area were greater in PH-treated plants in comparison to an untreated control, by 35, 24, and 26%. Increase in nitrogen assimilation and pigment synthesis in response to PH treatments has been also attributed to auxin as well as gibberellin-like activities (Ertani et al., 2009; Nardi et al., 2009; Colla et al., 2014). The biostimulant effects of low molecular size peptides and free amino acids have been also demonstrated by Matsumiya and Kubo (2011), who reported an increase of 25% in fresh weight of *Brassica rapa* with the addition of 12 mg-peptides/kg soil of degraded soybean meal products. The growth of eggplant, tomato and Indian mustard were also promoted by the addition of plant growth promoting peptides derived from soybean (Matsumiya and Kubo, 2011). In addition to stimulation of fresh weight, application of degraded soybean meal products increased the root hairs characteristics (number and length) of *Brassica oleracea* L., *Lactuca sativa* L., *Trifolium incarnatum* L., and *Gypsophila elegans* M. Bieb., thus favoring the uptake of water and nutrients via an increase in root surface. Similarly, Ugolini et al. (2015), reported that a sunflower meal hydrolysate containing free amino acids, with auxin-like but not gibberellins-like activity, stimulated root elongation, and increased transplanting success and crop productivity, indicating that this product could be an effective biostimulant in the agricultural field.

Protein hydrolysates have also been demonstrated to improve the productivity of ornamental plants. Application of two PHs derived from animal epithelia and alfalfa increased the diameter of flower buds, leaf area, stem quality and root biomass of lily (*Lilium longiflorum* Thunb. *× Lilium elegans* Thunb.) in comparison to untreated plants (De Lucia and Vecchietti, 2012). In contrast to studies demonstrating positive effects of PHs on plants, other experimental studies have found that foliar or root application of PHs has been minimal or non-significant (Kirn et al., 2010; Kunicki et al., 2010; Gajc-Wolska et al., 2012; Grabowska et al., 2012). For example, in these studies application of the animal-derived PH product ‘Siapton,’ had no effect on yield of endive, spinach, carrot and okra grown under open field conditions. The contrasting results may be due to the different origin of PHs (animal or vegetal origin), PH production process (chemical or enzymatic hydrolysis), plant species, rates of application and environmental conditions.

The amelioration of abiotic stress effects is the most commonly referred to benefit in relation to the use of biostimulants, since 60–70% of the yield losses in agriculture are estimated to be attributable to abiotic stresses (Rouphael et al., 2017c; Yakhin et al., 2017). Application of ‘Stressal,’ a commercial formulation of animal-derived PH, alleviated salt stress on persimmon (*Diospyros kaki* L.f.) by lowering chloride uptake and translocation to aerial parts, thus reducing leaf necrosis symptoms (Visconti et al., 2015). Greater tolerance to salt stress was associated with the composition of the PH, particularly compatible solutes such as proline and glycine betaine. When hydrolysate-based biostimulants from alfalfa containing triacontanol as well as inodole-3-acetic acid, were applied to maize under high salinity conditions, plants were better able to withstand salinity stress (Ertani et al., 2011). Under saline stress conditions, biostimulant-treated plants exhibited higher potassium and proline concentrations than untreated controls. In a similar experiment by the same authors, an alfalfa hydrolysate applied to maize grown in soilless culture under saline conditions also improved plant biomass and increased leaf proline, phenylalanine ammonia-lyase activity as well as gene expression relative to salt-stressed controls (Ertani et al., 2013). Lucini et al. (2015) showed that substrate drench, and to a higher extent foliar spray plus substrate drench applications of a plant-derived PH biostimulant product ‘Trainer^®^,’ helped plants maintain higher photochemical activity of the photosystem II, and obtain better nutritional status in lettuce (*Lactuca sativa* L.) shoot tissues under 25 mM NaCl, resulting in greater crop performance. The authors concluded that the potential for plants to withstand salinity stress in response to PH treatment, involved processes related to oxidative stress mitigation, change of hormonal balance, as well as production of secondary metabolites including glucosinolate, sterols and terpenes. In a similar experiment, Rouphael et al. (2017a) reported that the combination of a microbial biostimulant product ‘Click Horto’ (containing endophytic fungi such as *Rhizophagus intraradices* and *Trichoderma atroviride*) in combination with PH ‘Trainer^®^,’ induced a significant increase in crop productivity. Positive effects were associated with an increase in antioxidant enzymes activities (CAT and GPX), chlorophyll biosynthesis and improved mineral composition, likely through a stimulation of root morphology traits like total root length and root density. Cerdán et al. (2013) also demonstrated that the application of PH containing amino acids, particularly those derived from plant origin, enhanced tomato seedling growth under alkaline conditions due to an increase in leaf and root Fe reductase activities when applied to roots.

The potential for PH application to minimize the negative effects of thermal stress in several vegetable crops and perennial ryegrass have also been highlighted by several authors (Marfà et al., 2009; Botta, 2013). In the first experiment to investigate this potential benefit, the application of hydrolysates coming from animal hemoglobin did not improve strawberry (*Fragaria × ananassa* Duch.) plant survival following cold stress, through some growth promotion was recorded under non-cold stress conditions (Marfà et al., 2009). In contrast, enhancing plant tolerance to sub- and supra-optimal temperature conditions was observed in lettuce and ryegrass (*Lolium perenne* L.) when the commercial biostimulant ‘Terra-Sorb foliar’ containing amino acids was applied (Botta, 2013). In these experiments, PH-treated lettuce plants subjected to three cold stress treatments exhibited higher fresh weight compared to untreated plants, along with higher stomatal conductance, thus improving productivity. Moreover, PH-treat ryegrass plants subjected to high temperatures (36°C) had improved photosynthetic efficiency, levels of chlorophylls and carotenoids over control plants.

Protein hydrolysates can also help plants to perform better under low nutrient availability through an increase of nutrient use efficiency. In fact, Colla et al. (2013) demonstrated that weekly foliar applications of ‘Trainer^®^,’ at a dose of 2.5 ml/L increased the yield, greenness readings (i.e., SPAD index) and N uptake of baby lettuce plants by 50%, 11% and 11%, respectively, under reduced nutrient solution concentration (10% of standard solution). Thus, application of PH-biostimulants could be considered an effective tool for obtaining high productivity with lower impact on the environment. Finally, according to scientific literature, some key amino acids (e.g., asparagine, cysteine and glutamine) and peptides (e.g., glutathione and phytochelatins), could play an important role in the tolerance of plants to a range of toxic and heavy metals (Cu, Zn, As, Cd, and Ni) through metal chelation and binding (Sharma and Dietz, 2009; Sytar et al., 2013).

### Quality of Fruits and Vegetables

Over the past 20 years demand for high quality fruits and vegetables has been on the rise, in response to growing interest of consumers in healthy eating (Kyriacou et al., 2016, 2017). As reported in the previous sections, PHs have been shown to trigger several physiological mechanisms under optimal and sub-optimal conditions, stimulating the production and accumulation of specific molecules and secondary metabolites (i.e., ascorbate, tocopherols, carotenoids, glucosinolates). These metabolites perform a crucial role in supporting plant growth under suboptimal soil and ambient conditions, moreover such molecules confer an added value in promoting human well-being and longevity (Erba et al., 2013).

A significant improvement in protein, total phenolics, flavonoids, as well as antioxidant activity was observed when banana plants where fertilized with feather degradation products containing both amino acids and peptides (Gurav and Jadhav, 2013). This data confirmed results of earlier studies, which found an increase in phenols in various plant species, with the addition of organic wastes (McGrath et al., 1994). In red grape (*Vitis vinifera* L.), application of enzymatically treated vegetable extract coming from agricultural wastes increased the total phenolic and anthocyanin concentration by 22 and 70% respectively, over control plants (Parrado et al., 2007).

Ertani et al. (2014) conducted a greenhouse pot experiment with the goal of assessing the effects of two rates of biostimulants, one derived from alfalfa plants (25 and 50 ml/L) and another from red grapes (50 and 100 ml/L), on nutraceutical properties of *Capsicum chinensis* L. Results of these studies indicated that green pepper fruits of PH-treated plants had high concentrations of chlorogenic acid, and antioxidant activity, whereas both alfalfa and red grape PH-treated red pepper fruits were highly enriched with capsaicin. High-resolution magic-angle spinning-nuclear magnetic resonance spectra of red pepper fruits indicated that there were high amounts of NADP^+^ in treated plants from both PH sources, while red grape-PH treatment improved glucose, ascorbate, thymidine and other high molecular weight species (Ertani et al., 2014).

In a greenhouse tomato trial, foliar applications of a legume-derived PH ‘Trainer^®^’ enhanced antioxidant activities, soluble solids, mineral composition (K and Mg) as well as bioactive molecules such as lycopene and ascorbic acid, thereby increasing the nutritional and functional quality of the tomato fruits (Rouphael et al., 2017b). Similar findings were also reported by Colla et al. (2017) in another greenhouse tomato trial using the same PH.

The beneficial effects of PH-biostimulant was also observed on the phytochemical profile of lemon balm (*Melissa officinalis* L.), an important aromatic plant (Mehrafarin et al., 2015). The authors reported that a foliar application of commercial formulations of Aminolforte and Fosnutren at 2 l/ha, increased contents of citronellal, neral, deltacadinene, germacrene, and geranial compared to untreated plants.

PH application has also been shown to reduce nitrates in leafy vegetables, which are noted for their high nitrate accumulation and potential to harm human health when provided excessive consumption of vegetable greens (Amr and Hadidi, 2001). For instance, Liu and Lee (2012) demonstrated that the application of mixed amino acids could substantially reduce nitrate accumulation in several leafy vegetables such as lettuce, rocket (*Eruca sativa* Mill.), Swiss chard (*Beta vulgaris* var. *cicla* L.) and spinach (*Spinacea oleracea* L.). A negative correlation was observed between the accumulation of nitrates in lettuce leaves and the application of Amino 16 (Tsouvaltzis et al., 2014). A reduction in nitrate accumulation with the use of single amino acids was also observed on hydroponically grown pack choi (*Brassica rapa* subsp. *chinensis* L.) (Wang et al., 2007). The potential of PHs in preventing the high concentration of nitrates could be attributed to the up-regulation of several metabolic pathways involved in nitrogen metabolism, in particular nitrite and nitrate reductase as well as glutamate synthase and glutamine synthetase activities (Calvo et al., 2014; Colla et al., 2015a).

## Effects on Microbiome

Plant-associated microbes are increasingly being recognized for their potential to improve plant fitness by altering physiological and development processes (Philippot et al., 2013). Many of these interactions occur in the rhizosphere, a narrow zone of soil that surrounds and is influenced by plant roots, or in the phyllosphere, which covers plant leaf surfaces. The organic molecules in PHs could be used as a source of carbon, nitrogen and/or energy by the microbes residing in these unique habitats. Consequently, alteration of the composition and activity of plant microbiomes by PH’s could be yet another mechanism responsible for the improvement in crop productivity by these products.

### The Plant Microbiome

Plant-associated microorganisms have successfully coevolved with their host and are now known to play a crucial role in both crop growth and ecosystem functioning (Turner et al., 2013; Marin et al., 2017). To highlight the dependence of a plant on its microbiota at all stages of development, the concept of what constitutes an individual plant was redefined and plants are now perceived as a “metaorganism” or “holobiont” (Bordenstein and Theis, 2015; Vandenkoornhuyse et al., 2015; Rosenberg and Zilber-Rosenberg, 2016) or, considering also the interactions with the environment and other organisms, as a “phytobiome” (Baltrus, 2017; Leach et al., 2017).

In nature, healthy and asymptomatic plants are not axenic organisms but host a complex microbial consortium comprising bacteria, fungi, protists and viruses, many of which interact with plants in various (beneficial, neutral or harmful) ways. These microbial communities can affect plant health and productivity (Berendsen et al., 2012; Berg et al., 2016); help the plant to overcome biotic or abiotic stresses (Vorholt, 2012; Bulgarelli et al., 2013); and prevent pathogen attack (Mendes et al., 2013). Recently, the use of high-throughput sequencing and microbial-specific databases have provided deep insights into the composition of above- and belowground compartments of various host plants, including *Arabidopsis thaliana* (Lundberg et al., 2012; Horton et al., 2014; Bai et al., 2015), barley (*Hordeum vulgare* L.; Bulgarelli et al., 2015), corn (Peiffer et al., 2013), grapevine (Zarraonaindia et al., 2015), lettuce (Williams and Marco, 2014), potato (*Solanum tuberosum* L.; İnceoğlu et al., 2011, 2012), tomato (Ottesen et al., 2013), rice (*Oryza sativa* L.; Knief et al., 2012), sugarcane (*Saccharum officinarum* L.; Yeoh et al., 2016), and soybean (Mendes et al., 2014). These studies have demonstrated that plants harbor different microbial communities specific for each organ and that there are conserved taxa that inhabit a given plant organ across multiple host species and environments (Müller et al., 2016). These studies indicate that the root microbiome of phylogenetically unrelated plant species is composed of only a few dominant phyla, mainly belonging to Proteobacteria, Actinobacteria, Bacteroidetes, and to a lesser extent, Firmicutes (Lundberg et al., 2012; Berg et al., 2016), whereas fungal communities appear to be subjected to greater variation, are more dependent on biogeography, plant species and compartment, and stochastic variations (Shakya et al., 2013). Consistent with this, Coleman-Derr et al. (2016) observed that geographic origin of the host was the major driving factor in fungal but not bacterial communities associated with cultivated and native agaves.

Bacteria also tend to be the most abundant microorganisms in phyllosphere communities. In particular, Proteobacteria, Firmicutes, and Actinobacteria often dominate the plant phyllosphere, with *Methylobacterium*, *Pseudomonas*, and *Sphingomonas* being among the most abundant genera at the leaf level in *A. thaliana*, soybean and grapevine (Delmotte et al., 2009; Zarraonaindia et al., 2015), and *Pseudomonas* and *Erwinia* (*Pantoea*) are the predominant taxa at the flower level, at least in grapevines (Zarraonaindia et al., 2015).

While the beneficial or detrimental effects of root-associated microbes have received considerable attention in recent years (Berendsen et al., 2012; Bulgarelli et al., 2013; Mendes et al., 2013; Philippot et al., 2013; Berg et al., 2016) the effects of epiphytes on plant health and productivity are not as well known. Like root-associated microbes, some epiphytic microbes have been demonstrated to promote plant growth via production of hormones (Wu et al., 2009; Ruzzi and Aroca, 2015) or synthesis of volatile organic compounds (VOCs), by biotransformation (Marmulla and Harder, 2014) or *de novo* biosynthesis (Schulz and Dickschat, 2007), that can have antimicrobial effects or serve as carbon sources for some microorganisms (Farré-Armengol et al., 2016). Plant pathogens can colonize the phyllosphere in the absence of any apparent infection (Vorholt, 2012), while other microbes such as some *Pseudomonas* and *Sphingomonas* species protect plants from pathogens by competing for limited nutrients, producing antibiotic compounds (Lindow and Brandl, 2003; İnnerebner et al., 2011; Ritpitakphong et al., 2016), and inducing systemic resistance (Conrath et al., 2006; Pieterse et al., 2012). Finally, the importance of the phyllospheric microbiota on the metabolic function of aromatic plant species has been recently analyzed in both *Sambucus nigra* L. and *Mentha piperita* Huds. del Rosario Cappellari et al. (2017) demonstrated that co-inoculation with selected *Pseudomonas* and *Bacillus* plant-growth promoting strains induced greater emissions of VOCs emission and synthesis of phenolic compounds in *M. piperita* plants. In contrast, Gargallo-Garriga et al. (2016) demonstrated that in *Sambucus nigra* L., suppression of phyllospheric microbial communities led to a decrease in several metabolites, such as citraconic acid, acetyl-CoA, isoleucine, as well as secondary compounds including terpenes and phenols.

In summary, a stable increase in plant productivity can be achieved if beneficial plant–microbiome relationships are established and maintained in the rhizosphere and phyllsophere. However, before this becomes a reality, greater understanding of factors that regulate these key plant-associated habitats is needed.

### Plant–Microbial Habitats

In 1904, Lorenz Hiltner first coined the term “rhizosphere” and theorized that microbes inhabiting this plant-soil interface likely play a role in plant nutrition, growth promotion and suppression of plant pathogens (Hartmann et al., 2008). One hundred years later, it is now commonly accepted that the rhizosphere is one of the most dynamic and biologically active environments on earth, and microbes residing in this habitat are crucial for maintaining plant health in natural and managed ecosystems (Berendsen et al., 2012). Study of the rhizosphere and ways to manipulate this critical plant–soil interface to benefit plants in agricultural and horticultural systems has now become a prominent area of research.

While recent studies indicate that the composition of rhizosphere microbial communities is highly diverse, microbial taxa inhabiting this environment clearly differ from bulk soil (Berendsen et al., 2012; Philippot et al., 2013). This is likely due to differences in physicochemical characteristics between these two habitats. For example, nutrient and water availability as well as soil pH are modified by the presence of plant roots, and these conditions are likely to be important factors in the type of microbial taxa that can thrive in this environment. Moreover, plants roots actively and passively release up to 40% of their photosynthetically derived carbon via root exudates, mucilage, and sloughed off root cells (Bais et al., 2006). Release of these compounds signal and provide support for rhizosphere microbial communities. Composition of these carbon compounds, as well as rhizosphere microbial community structure, vary given plant species and genotype, root morphology, plant development and maturation, and even location on the root (Berg and Smalla, 2009). Thus, while soil type, management history and climatic conditions arguably play a key role in shaping rhizosphere microbial communities since they influence the composition of microbial taxa available for colonization, it is clear that plants recruit specific microbial taxa and selectively shape this community via composition of these carbon compounds (Berg and Smalla, 2009).

Carbon compounds released from plant roots are made up of a mixture of low molecular weight (amino acids, organic acids, sugars and phenolics) as well as high molecular weight compounds (polysaccharides and proteins) (Badri et al., 2009). In particular, amino acids are the second most abundant compound released from plant roots, and the potential for rhizospheric microbes to utilize these compounds is thought to be a key characteristic of microbes residing in this habitat. In fact, over 80% of rhizosphere microbes have been found to possess this capability (Moe, 2013). Soil microbes have also been shown to specifically chemotax toward amino acids in root exudates (Nelson, 2004), another essential trait for rhizopshere colonization (de Weert et al., 2002), and the half-life of these compounds in soil averages just 1–6 h (Jones and Kielland, 2012). Amino acids are thought to provide an important food and energy source for microbes, as well as a mechanism to help modify various stress responses (Moe, 2013). Because of their importance, it has even been theorized that microbes produce compounds that help them to compete for amino acids with plants. For example, production of 2,4-diacetylphloroglucinol (DAPG), a compound commonly produced by many *Pseudomonas fluorescens* strains and frequently cited for its antagonistic activity toward plant pathogens, appeared to block amino acid influx by plants (Phillips et al., 2004).

The presence of amino acids in the rhizosphere has been shown to affect many key processes that help microbes survive in the rhizosphere and could also be indirectly affecting plants. For example, amino acids are major determinants of the synthesis and activity of auxin phytohormones like IAA (Staswick, 2009). Many rhizosphere-dwelling bacteria have been shown to synthesize IAA, with estimates that up to 80% possess this trait (Patten and Glick, 1996). Another key feature of microbial taxa inhabiting the rhizosphere is the ability to produce and reside in biofilms (Danhorn and Fuqua, 2007). Biofilms help microbes withstand environmental stress caused by pH, salt or toxic compounds produced by plants and other microbes. They also protect microbes from grazing by protozoa and facilitate horizontal gene transfer. Most importantly, they help microbes maintain critical mass for periods sufficient to initiate consortial metabolism that single cells cannot accomplish effectively. For example, the products of consortial metabolism include metabolites and exoenzymes important in degradation of organic matter, biocontrol activity and pathogenesis. Amino acid composition has been shown to be a key factor in biofilm formation by some microbial taxa, as well as disassembly in others (Kolodkin-Gal et al., 2010).

Because amino acids make up a significant component of PHs, it may be possible to modify the composition of these products and thereby alter rhizosphere microbial community structure and activity. For example, using soil dilutions, Halvorson (1972) found dramatic difference in the potential for soil microbes to utilize individual amino acids. The highest colony counts were observed in selective media containing threonine (79.2%), aspartate (70.8%), and glutamate (66.7%), whereas cysteine (8.1%) and tryptophan (7.7%) showed the lowest colony counts. Differences in preference among individual microbial taxa for specific amino acids have also been noted (Moe, 2013), providing further support for the theory that PHs could be specifically formulated to support specific microbial taxa.

The phyllosphere has received much less attention than the rhizosphere, though recent studies have begun to shed light on this important plant-microbial habitat. Unlike the rhizosphere, which is thought to favor copiotrophic organisms that can rapidly utilize labile carbon compounds released from plant roots, the phyllosphere is thought to be an oligotrophic environment, with few available nutrients, especially carbon (Vorholt, 2012). The phyllosphere is also expected to be a more ephemeral or short-lived habitat in comparison to the rhizosphere, with the microbes in this habitat subjected to more stressful environmental conditions. For example, phyllosphere microbes may be exposed to ultraviolet (UV) radiation as well as extreme drought due to the waxy cuticle covering plant leaves, which prevents water loss (Vorholt, 2012). At the same time, phyllosphere microbes are also subjected to intense rainfall events. While overall species richness in the phyllosphere is high, diversity compared to the rhizosphere and bulk soil is much lower, with over 70% of phyla characterized as Alphaproteobacteria, and the rest assigned primarily to Gammaproteobacteria, Bacteriodes and Actinobacteria (Vorholt, 2012). Consequently, manipulating the phyllosphere microbiome using PHs could prove easier than trying to manipulate the rhizosphere microbiome, since there are likely to be fewer microbial taxa inhabiting this environment and nutrients are scare.

Like the rhizosphere, distribution of phyllosphere microbes have been found to be highly heterogeneous, with microbes often residing in aggregates located near sites of nutrient leakage from plants such as the stomata and base of trichomes (Lindow and Brandl, 2003; Vorholt, 2012). Microbial composition has also been found to vary given plant species and genotype, development stage, as well as location on the leaf, which could be due to differences in surface appendages and composition of plant leachates (Lindow and Brandl, 2003; Vorholt, 2012; Ortega et al., 2016). Key traits expected to be essential for supporting microbial life in the phyllosphere include chemotaxis, and the ability to produce biofilms as well as pigments that aid in UV tolerance (Lindow and Brandl, 2003; Vorholt, 2012). Like rhizosphere microbes, the ability to withstand and produce antagonistic compounds is also expected to be essential to the ability of individual taxa to compete for nutrients and space. In particular, a high proportion of microbes isolated from the phyllosphere (up to 58%) were able to inhibit pathogen growth by production of VOCs (Ortega et al., 2016). The potential for microbes to synthesize IAA appears to be widespread among phyllosphere microbes and could be an important factor in facilitating colonization and helping microbes withstand drought stress (Lindow and Brandl, 2003). There is also evidence that microbial synthesis of IAA could increase the availability of nutrients by loosening cell walls, thereby releasing saccharides from plants (Fry, 1989; Lindow and Brandl, 2003; Vorholt, 2012). Finally, microbial production of surfactants also appears to be an essential trait for helping phyllosphere microbes to withstand drought stress (Lindow and Brandl, 2003; Vorholt, 2012).

### Protein Hydrolysates Effects on the Plant Microbiome

In response to increasing awareness of the plant microbiome and identification of specific microbial taxa than can benefit plants, scientists have begun to investigate whether PHs are indirectly affecting plant growth by altering these communities. To our knowledge, the only study to date that has specifically tested this hypothesis in soil is the study by Tejada et al. (2011), which correlated application of biostimulants with alterations in soil microbial community structure and greater soil microbial activity with improved plant establishment on degraded soils. Soil microbes are well known for their potential to produce extracellular enzymes that aid in decomposition of organic matter, producing compounds that could directly affect plants. For example, as noted above, an alkaline protease produced by *Bacillus circulans* HA12 has been used to produce a bioactive peptide called ‘root hair promoting peptide’ in a PH derived from soybeans (Matsumiya and Kubo, 2011). Composition of PHs resulting from the type of hydrolysis treatment used as well as the original feedstock is likely to affect microbial activity and corresponding plant benefits. Of the four biostimulants evaluated in the study by Tejada et al. (2011), the amendment derived from rice bran extract had the greatest effect on soil microbes and vegetal cover. The authors concluded that this was likely due to the fact that the rice bran extract contained the highest amount of protein and percentage of peptides under 3kDa, and the low molecular weight of these compounds could easily be assimilated by microbes. Furthermore, the authors suggested that a lower fat content in biostimulants could also favor nutrient and peptide absorption by microbes. Additional studies investigating relationships between PHs, soil and root microbiomes, and plant productivity are needed.

A few scientists have begun to try and untangle the complex relationships between biostimulant formulations, phyllosphere microbial community structure and activity, and plant health. For example, using a culture-dependent approach, Luziatelli et al. (2016) determined that a PH-based biostimulant product derived from a legume (‘Trainer^®^’) and another product derived from tropical plant extracts (‘Auxym^®^’), altered phyllosphere microbial community diversity and increased lettuce growth and leaf chlorophyll content. Many of the microbes isolated from lettuce leaves subjected to foliar applications of these products, most notably isolates from the genera *Pantoea*, *Micrococcus*, and *Acinetobacter*, had the potential to solubilize phosphorous and produce indole acetic acid (IAA). Moreover, all *Bacillus* strains isolated from lettuce leaves exhibited strong inhibitory activity against two key plant pathogens (*Fusarium oxysporum* and *Erwinia amylovora*), and isolates of *Pantoea*, *Micrococcus* and *Pseudomonas* were active against *E. amylovora*. Results of this study indicate that alteration of the phyllospere microbial community not only stimulates plant growth, but could also help plants withstand pathogen stress.

Hydrolysates derived from casein and soybeans have previously been demonstrated to elicit grapevine defense mechanisms and suppress downy mildew, caused by *Plasmopara viticola* (Lachhab et al., 2014), but it was unclear whether this was due to direct induction of plant defense responses or an indirect effect from modification of phyllosphere microbial communities. Cappelletti et al. (2016) sought to answer this question by studying the effects of a protein derivative on downy mildew in grape in the absence (axenic conditions) and presence of phyllopshere microorganisms (protected cultivation). Results of these studies confirmed that the protein derivative could stimulate plant defense responses and reduce downy mildew in grape, and the authors concluded that multiple mechanisms of action were likely involved in the suppressive effects observed. For example, while induction of some defense genes were observed under axenic conditions, others were expressed only in the presence of phyllosphere microbial communities indicating that biocontrol activity of these microbes likely played a role in downy mildew suppression. Many of the microbial genera isolated from grapevine leaves in this study, including *Exiguobacterium*, *Pseudomonas*, *Serratia*, and *Lysobacter* species have previously been found to contribute to biocontrol activity via multiple strategies including competition for space and production of antagonistic compounds.

These studies provide evidence that PHs can modify microbial community structure and activity, and such changes could contribute to some of the beneficial effects observed after applying these products. Moreover, they provide tantalizing support for the hypothesis that these products could someday be specifically formulated to support beneficial plant-microbial relationships and further enhance plant productivity. In support of this hypothesis, Rouphael et al. (2017a) recently demonstrated that tolerance to alkalinity and salinity of lettuce plants could be improved by combining a PH with a microbial-based biostimulant containing *Rhizophagus intraradices* (an arbuscular mycorrhizal fungus, AMF) and *Trichoderma atroviride* (a filamentous fungus that functions as biocontrol agent). The effect of the combined application of the PH and fungi on plant growth was attributed to several factors, including increase in root surface area, greater chlorophyll synthesis and proline accumulation.

## Conclusion

Protein hydrolysates have great potential to improve crop performance, especially under environmental stress conditions. Root applications of PHs have been shown to be beneficial by improving nutrient use efficiency, enhancing nutrient availability, root growth, nutrient uptake and assimilation in several crops. Moreover, foliar and root (substrate drench) applications of PHs exhibit hormone-like activities (especially auxin-like and gibberellin-like activity) leading to stimulation of seed germination, plant growth, fruit set and enlargement. PHs not only increase yield but also improve some quality parameters such as fruit size, skin color, soluble solids, and antioxidant contents. Moreover, PHs have also great potential to reduce nitrate accumulation in leafy vegetables such as lettuce, spinach and rocket. However, mechanisms regulating the beneficial effects of PHs on plants are not completely understood and only recently, thanks to use of ‘omics’ sciences, is it becoming possible to clarify specific modes of action.

Recent studies have provided evidence that PHs can affect plant microbiomes and some of the benefits derived from these products might be due in part to changes in the composition and activity of these plant-associated communities. Combining PHs with specific microbial taxa that are well known for their potential to help plants acquire nutrients and withstand biotic and abiotic stress has been demonstrated to further enhance plant benefits. Someday it might be possible to build on the results of these studies by specifically formulating PHs to enhance the abundance and activity of beneficial microbes naturally inhabiting plant compartments, and/or develop consortia of fungi and bacteria that can be applied in combination with PHs to improve plant performance. For example, it has been reported that plant productivity is directly related to evenness (relative abundance) of members of the microbiome (Wilsey and Potvin, 2000), and increasing microbial biomass and/or diversity can enhance pathogen- or disease-suppressiveness (Larkin and Honeycutt, 2006). If microbial taxa that are evolutionarily adapted to particular host plants can be identified and applied in concert with PHs to support their colonization and survival on plants, these consortia could reduce the time required for the microbiome to achieve niche saturation and competitively exclude pathogens. The strategy of using plants as selective agents to improve beneficial microbial functions has the major advantage of not requiring any change in infrastructure or management.

## Future Directions

Further maximizing the beneficial effects of PHs will require a better mechanistic understanding of how the rate, timing of application and composition of individual products specifically alters plant physiological processes. Moreover, there is growing consensus that small size peptides play an important role in the biological activity of PHs. However, only few bioactive peptides have been characterized. Therefore, more studies are necessary to discover the signaling peptides, which are responsible for the biostimulant activity of PHs. These findings may also help to make the PH production process more efficient in producing bioactive peptides.

Effectively manipulating plant microbiomes with PHs will require additional research to answer questions such as: how do individual microbial taxa respond to specific amino acids and other compounds in PHs; what is the optimal dose, time and mode of application to support specific microbial taxa that improve plant fitness; how much will plant species, genotype and the environment affect these relationships; can PHs be formulated with specific compounds to better support colonization and survival of microbial inoculants; and, will PHs need to be combined with inoculants that contain a microbial consortia with synergistic traits, thus providing more consistent effects? Application of new ‘omics’ sciences and high-throughput phenotyping platforms will aid in these studies, though partnerships between academic researchers and private industry will be required due the high costs of these studies. At the same time, further research investigating the effects of growing conditions on the interactions between PH formulation, plant species, developmental stage, application rate, microbiomes, etc., are also needed.

While there seem to be more questions than answers at this point in time, results of the few studies that have attempted to start to tease apart the complex relationships between PHs, the plant microbiome and changes in plant physiological processes suggest that altering these relationships will be possible and will be well worth the effort.

## Author Contributions

GC coordinated the review and he wrote many parts of the article. LH wrote part of the review dealing the effects of protein hydrolystes on microbiome and she contributed to improve the article. MR wrote part of the review dealing with the microbiome and he contributed to improve the article. MC wrote part of the review dealing with germination and seedling growth and she contributed to improve the article. PB wrote part of the review dealing with protein hydrolysate effects on plant growth. RC wrote part of the review dealing with chemical characteristics of protein hydrolysates. YR wrote many parts of the article especially those dealing with the effects of protein hydrolysates on yield and quality and he contributed to improve the article.

## Conflict of Interest Statement

The authors declare that the research was conducted in the absence of any commercial or financial relationships that could be construed as a potential conflict of interest. The reviewer AQ and handling Editor declared their shared affiliation.
